# Monocytes and pyrophosphate promote mesenchymal stem cell viability and early osteogenic differentiation

**DOI:** 10.1007/s10856-021-06639-y

**Published:** 2022-01-15

**Authors:** Sara Svensson, Michael Palmer, Johan Svensson, Anna Johansson, Håkan Engqvist, Omar Omar, Peter Thomsen

**Affiliations:** 1 Department of Biomaterials, Institute of Clinical Sciences, Sahlgrenska Academy, University of Gothenburg, Gothenburg, Sweden; 2 Department of Engineering Sciences, Applied Materials Science Section, Uppsala University, Uppsala, Sweden; 3 Department of Statistics, Umeå School of Business, Economics and Statistics, Umeå University, Umeå, Sweden; 4 Department of Biomedical Dental Sciences, College of Dentistry, Imam Abdulrahman bin Faisal University, Dammam, Saudi Arabia

**Keywords:** Cell-cell communication, Gene expression, Monocytes, MSC, Osteoinduction

## Abstract

Pyrophosphate-containing calcium phosphate implants promote osteoinduction and bone regeneration. The role of pyrophosphate for inflammatory cell-mesenchymal stem cell (MSC) cross-talk during osteogenesis is not known. In the present work, the effects of lipopolysaccharide (LPS) and pyrophosphate (PPi) on primary human monocytes and on osteogenic gene expression in human adipose-derived MSCs were evaluated in vitro, using conditioned media transfer as well as direct effect systems. Direct exposure to pyrophosphate increased nonadherent monocyte survival (by 120% without LPS and 235% with LPS) and MSC viability (LDH) (by 16–19% with and without LPS). Conditioned media from LPS-primed monocytes significantly upregulated osteogenic genes (ALP and RUNX2) and downregulated adipogenic (PPAR-γ) and chondrogenic (SOX9) genes in recipient MSCs. Moreover, the inclusion of PPi (250 μM) resulted in a 1.2- to 2-fold significant downregulation of SOX9 in the recipient MSCs, irrespective of LPS stimulation or culture media type. These results indicate that conditioned media from LPS-stimulated inflammatory monocytes potentiates the early MSCs commitment towards the osteogenic lineage and that direct pyrophosphate exposure to MSCs can promote their viability and reduce their chondrogenic gene expression. These results are the first to show that pyrophosphate can act as a survival factor for both human MSCs and primary monocytes and can influence the early MSC gene expression.

**Figure:**
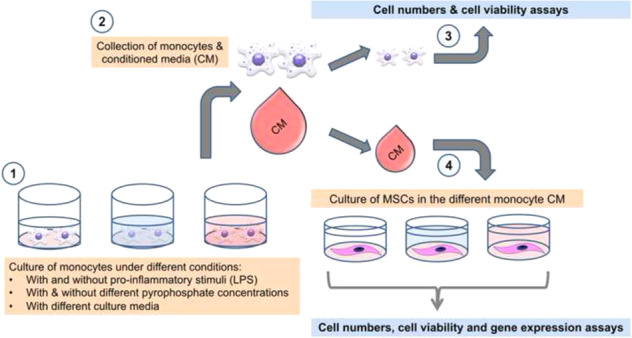
Graphical abstract

Graphical abstract

## Introduction

Bone tissue has a remarkable ability to regenerate following injury. However, malunion or nonunion still occurs in more than 10% of traumatic injury cases [1], especially when the degree of injury exceeds the body’s ability to repair or when the healing is compromised. Currently, the most promising pro-regenerative tools for bridging large defects and improving the rate of bone regeneration are materials (i.e., tissue scaffolds or granules) and biochemical signals that stimulate the recruitment and/or differentiation of stem cells and osteoprogenitors (e.g., growth factors, such as BMP-2, or bioactive ions, such as calcium and strontium) [2, 3]. The bone regenerative effects of such natural and synthetic cues are commonly judged by the response of osteoblastic cell lines in vitro or the degree of bone formation in vivo. On the other hand, the role of other cell types and biological processes, such as immune cells and inflammation, in mediating potential material effects remains much less explored.

Inflammatory cells play a major role in bone tissue healing and regeneration after trauma. Circulating monocytes are actively recruited to an injury site [1, 4] where they differentiate into macrophages that recruit and activate multiple cell types. Monocytes/macrophages express and secrete biological mediators that recruit mesenchymal stem cells (MSCs) [5] and osteoblasts [6]. Moreover, progenitors from the monocytic lineage can directly differentiate into bone-resorbing osteoclasts [6] and can also polarize towards pro-inflammatory (M1) or pro-regenerative (M2) macrophage phenotypes [7–9]. In addition to their role during the acute healing phase, it has been suggested that signals secreted by macrophages modulate the regenerative process even after the initial inflammatory response is resolved [8, 9]. It has been shown in vitro that conditioned media (CM) from macrophages cultured on smooth and microporous titanium surfaces, under different stimulation conditions, convey differential osteogenic effects on recipient MSCs [10]. Interestingly, the modest effect of the surface properties communicated by macrophages was surpassed by the classical activation of M1 macrophages by lipopolysaccharide (LPS) [10]. Specifically, proinflammatory M1 macrophages induced osteogenic gene expression in recipient MSCs, but IL-4-stimulated (M2) macrophages did not [10]. In contrast, another study concluded that material-induced M2 macrophages stimulate the proliferation and differentiation of MSCs, leading to improved vascularization and angiogenic gene expression [11].

In vitro studies have suggested that specific phases of calcium phosphates promote inflammatory, immunomodulatory and osteogenic responses from monocytes/macrophages and/or MSCs [11–13]. Calcium pyrophosphate, an insoluble calcium phosphate that elicits a strong inflammatory response [13, 14], has been shown to stimulate osteogenic gene expression in vitro [15] and bone formation in vivo [16, 17]. With respect to inflammatory cells, pyrophosphate crystals induce potent expression and secretion of proinflammatory cytokines (IL-1β, IL-6, and IL-8) via activation of the MAPK-dependent NF-κB pathway [13]. The same pathway is well recognized to mediate LPS-induced expression and secretion of major proinflammatory cytokines by macrophages [18, 19]. Interestingly, inflammatory cells can also directly regulate endogenous pyrophosphate production and localization via proinflammatory cytokine-mediated expression of the pyrophosphate generating, transporting and digesting (phosphoesterase) proteins ENPP1, ANKH, and ENT 1 [20, 21]. However, it is unclear whether the macrophage response is affected by pyrophosphate concentration and whether the pyrophosphate-induced effects on macrophages are influenced by the presence of proinflammatory mediators, such as LPS.

Pyrophosphate ions are naturally transported out of osteoblasts and osteogenic progenitors (MSCs) to regulate mineralization and prevent pathological calcification of the extracellular matrix [20, 22, 23]. In contrast, when osteogenic cells encounter pyrophosphate-containing surfaces (i.e., coatings or cell scaffolds), their response is assumed to differ from that observed when contacting naturally formed nano- and micron-scale precipitants, which are known to elicit a vigorous inflammatory condition in joints (i.e., calcium pyrophosphate deposition (CPPD) in pseudogout) [24, 25]. Instead, pyrophosphate-containing materials [17] and coatings [26, 27] have been shown to promote osteogenic effects. Recently, pyrophosphate-containing bioceramics were shown to considerably promote bone regeneration in osseous and nonosseous sites [28]. Histological evidence revealed that the pyrophosphate-containing bioceramic induces the differentiation of MSCs to osteoblastic cells in parallel to macrophage accumulation in the vicinity of the implant and bone-forming osteoblasts. Therefore, it is important to explore whether exposure to pyrophosphate has a direct effect on MSCs or whether the pyrophosphate effect on MSCs is indirectly mediated via macrophages.

When evaluating biomaterials, although single cell type in vitro assays provide simple screening systems, they are far from representing the complex in vivo milieu [29]. Attempts have been made to mimic the 3-dimensional in vivo healing environment around implants, using, for instance, explanted [30] or tissue-engineered [31] bone in combination with regenerative stem cells. Moreover, conditioned media (CM) transfer [10, 32] and co-culture [33, 34] in vitro studies have provided mechanistic insight into the role of cell-cell communication in the cellular response to the material properties. Soluble factors and signals conveyed by inflammatory cells to stem cells are of particular interest.

In the present study, the effects of LPS and disodium pyrophosphate (PPi) on primary human monocytes and osteogenic gene expression in human adipose-derived MSCs were evaluated. The aim was to determine whether different concentrations of pyrophosphate influence macrophage and MSC responses and whether the priming of MSCs with LPS or LPS-treated monocyte conditioned medium enhances osteogenic gene expression in MSCs. The null-hypothesis is that LPS and/or PPi neither influence the human monocyte cell number and viability nor convey any direct or indirect effects on cell number, viability and early osteogenic gene expression of human MSCs.

## Materials and methods

### Pyrophosphate

A 50 mM sodium pyrophosphate stock solution was prepared by dissolving sodium pyrophosphate (H_2_Na_2_O_7_P_2_; Sigma-Aldrich, Steinheim, Germany) in deionized water, neutralizing with 2.0 M sodium hydroxide (NaOH) and passing through a 0.22 μm sterile filter. The stock solution was prepared fresh prior to each treatment.

### Cell culture

Buffy coats were purchased from the Blood Central (Sahlgrenska University Hospital, Sweden). The buffy coats were received after deidentification with respect to name, gender and age by the Blood Central. Primary human monocytes were isolated from buffy coats of five anonymous healthy blood donors using Ficoll separation followed by negative selection on a magnetic column (MACS; Miltenyi Biotec, Bergisch Gadbach, Germany; viability >98%). May-Grünewald Giemsa-stained cytospin slides showed 97.4 ± 0.3% monocyte purity, with lymphocytes being the primary source of contamination. Monocytes were seeded into tissue culture-treated 6-well polystyrene plates (Falcon™, BD Biosciences, San Jose, CA, USA) at a concentration of 0.5 × 10^6^ cells/mL in a total of 5 mL Dulbecco’s modified Eagle’s medium-low glucose (DMEM-LG; Lonza, Bornem, Belgium) supplemented with 10% fetal bovine serum (Gibco, UK), 1% L-glutamine (2 mM; Gibco) and 1% pencillin/streptomycin (PEST; Sigma-Aldrich, Saint Louis, MO, USA). After overnight incubation (37 °C, humidified atmosphere with 5% CO_2_), the medium was exchanged to a low-serum variant (1%), and the cells were treated with the following concentrations of pyrophosphate (PPi): 0 μM (PPi0), 50 μM (PPi1), 100 μM (PPi2) or 250 μM (PPi3) with or without 10 ng/mL lipopolysaccharide (LPS; Sigma-Aldrich), yielding a total of eight different environments (two wells per environment). The cells were incubated in the medium for 48 h, yielding monocyte-conditioned medium (CM). In parallel, control medium (CtrM) was produced by incubating in an identical set of 6-well plates using the same stimulatory conditions but without cells. In addition, medium to be used for direct stimulation of MSCs was placed in a flask in the incubator, later yielding direct effect medium (DEM). Monocyte-CM and CtrM were transferred into 15 mL tubes and centrifuged (500 g, 10 min), after which the supernatants from the same conditions were pooled (total 10 mL).

**Fig. 1:**
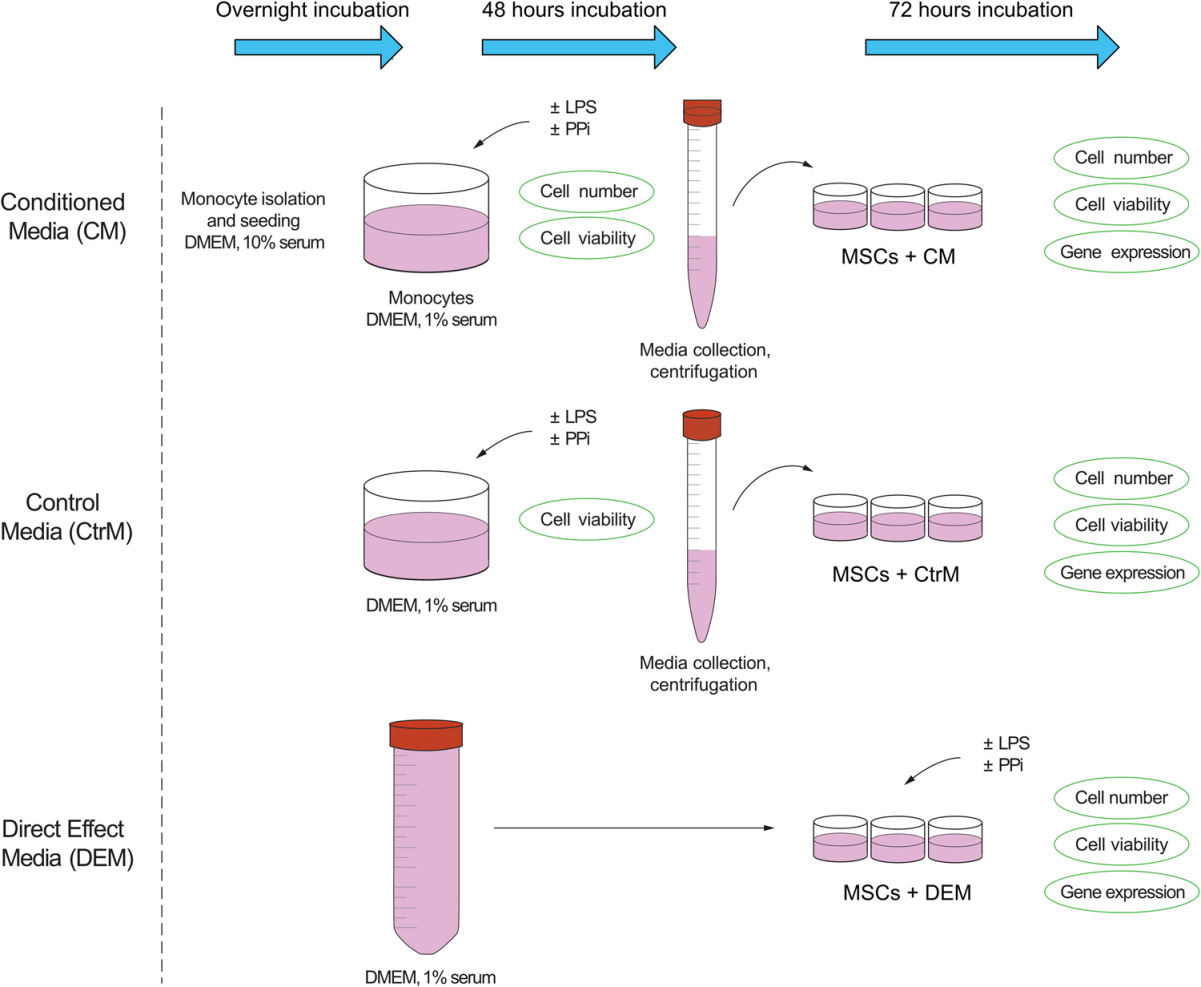
Schematic drawing of the experimental outline. Monocytes isolated from buffy coats were seeded into 6-well plates and incubated overnight. The medium was changed, and monocytes were exposed to varying concentrations of pyrophosphate (PPi) in the presence or absence of LPS for 48 h. The medium was withdrawn, centrifuged, and added to MSCs for 72 h prior to analysis. In parallel, control media (CtrM) – without monocytes – with the addition of PPi and LPS were incubated for 48 h before centrifugation and exposure to MSCs. In a third track, medium was incubated for 48 h before addition to MSCs with additional direct exposure of MSCs to PPi and LPS, referred to as direct effect media (DEM)

Human adipose-derived mesenchymal stem cells (ATCC #63557082), passage 5, were seeded into tissue culture-treated 24-well polystyrene plates (Falcon™) at a concentration of 36,000 cells/mL in 1 mL expansion medium containing DMEM-LG supplemented with 10% FBS, 1% L-glutamine (2 mM), 1% PEST and 0.01% fibroblast growth factor (FGF; Life Technologies, UK). After 48 h of culture, the medium was exchanged with CM, CtrM or DEM. The DEM wells were treated with the following PPi concentrations: 0 μM (PPi0), 50 μM (PPi1), 100 μM (PPi2) or 250 μM (PPi3) with or without 10 ng/mL LPS. After 72 h of incubation, the MSCs were harvested. The outline of the in vitro experiments is presented in Fig. 1.

### Cell number

Quantification of adherent and supernatant monocytes and MSCs (analyzed in duplicate samples) was performed using the Nucleocounter^®^ system (ChemoMetec A/S, Allerød, Denmark). Briefly, equal amounts of lysing buffer A and stabilization buffer B (provided with the system) were added to each sample; vortexing was performed after each addition and prior to measurement. The samples were loaded in a Nucleocassette^™^ containing propidium iodide, and the cell nuclei were automatically counted. For viability measurements of purified monocytes, an additional cell sample without the addition of lysis buffer was analyzed to detect dead cells in the sample.

### Cell viability

Cell viability was assessed in centrifuged medium (300 g, 5–10 min) in duplicate samples by analyzing the lactate dehydrogenase (LDH) content (C-laboratory, Sahlgrenska University Hospital, Gothenburg, Sweden). The detection limit of the instrument was 0.17 μKatal/L. Values below the detection limit were set at 0.16 μKatal/L.

### Gene expression

Total RNA was extracted from adherent MSCs in triplicate samples after the 72 h incubation period with mCM, CtrM or DEM. Cells were carefully washed with RNase-free PBS (HyClone, GE Healthcare Life Science, Logan, UT, USA), lysed in 350 μL buffer RLT (Qiagen, Hilden, Germany) supplemented with dithiothreitol (DTT; Sigma-Aldrich) during vortexing (60 s) and then frozen at −80 °C before on-column RNA isolation using an RNeasy® micro kit including DNase treatment (Qiagen). RNA concentrations were measured with a Pearl NanoPhotometer™ (Implen GmbH, Munich, Germany), and RNA quality was determined for selected samples using an Agilent RNA 6000 Nano Kit and an Agilent 2100 Bioanalyzer (Agilent Technologies, Foster City, CA, USA).

Total RNA, normalized to 20 ng/μL, was converted to cDNA using a TATAA Grandscript cDNA Synthesis Kit (TATAA Biocenter AB, Gothenburg, Sweden) in 10 μL reactions. Samples were diluted 15x, and real-time RT-qPCR analysis was performed in duplicate 10 μL reactions. Samples and mastermix prepared from TATAA SYBR® GrandMaster Mix (TATAA Biocenter AB) and primers (400 nM final concentration) were pipetted using the Eppendorf epMotion 5070 robotic system (Eppendorf, Hamburg, Germany) and run on the LightCycler^®^480 System (Roche Applied Science, Penzburg, Germany). The analyzed genes were related to the following: osteogenic differentiation: *alkaline phosphatase* (*ALP*), *bone morphogenetic protein 2* (*BMP-2*), *type I collagen alpha* (*COL1A1*), *runt-related transcription factor 2* (*RUNX2*), *osteopontin* (*OPN*), *transforming growth factor beta* (*TGF-β*); cell proliferation: marker of proliferation Ki-67 (*KI67)*, *proliferating cell nuclear antigen* (*PCNA*); cell death: *caspase 3* (*CASP3)*, tumor protein p53 (*P53)*; and chondrogenic- or adipocyte differentiation: *SRY-box transcription factor 9 (SOX9)* and *peroxisome proliferator-activated receptor gamma* (*PPAR-γ*). *Tyrosine 3-monooxygenase /tryptophan 5-monooxygenase activation protein zeta* (*YWHAZ*) was identified as the best reference gene in the TATAA reference gene panel as provided in the Online Resource 1 (Supplementary Table S1) based on analysis in GenEx software version 6 (MultiD Analyses AB, Gothenburg, Sweden) using both the geNorm and NormFinder algorithms. Raw data were analyzed on LightCycler®480 Software, Version 1.5 (Roche Applied Science) and processed in GenEx using the relative comparative Cq method. ValidPrime (TATAA Biocenter AB) was used for the detection and correction of contaminating genomic DNA.

### Statistical modeling and analysis

The experiment was analyzed in a series of MANOVAs in blocks of responses that were connected, i.e., bone-related gene expression (*ALP*, *BMP-2*, *COL1A1*, *RUNX2*, *OPN* and *TGF-β*), cartilage- or adipose-related gene expression (*SOX9* and *PPAR-γ)*, proliferation-related gene expression (*KI67*, *PCNA*), death-related gene expression (*CASP3*, *P53*), MSC cell numbers (adherent + supernatant cells) and viability, and monocyte cell numbers and viability. In MANOVAs related to MSCs, the model was defined by the factors LPS (with or without), material (PPi0, PPi1, PPi2, PPi3), the condition (CM, CtrM, DEM) and the interaction LPS×condition. In the MANOVA related to the monocytes, only the factors LPS and material were used. Individual factor-level effects were analyzed using estimated marginal means with Bonferroni correction. Further details on MANOVA results and associated complementary ANOVA analyses are found in the Online Resource 1 (Supplementary Table S2). Residual analyses were carried out to check the model assumptions. If the assumption of homoelasticity was doubtful, a sensitivity analysis using bootstrapping was carried out. The data were statistically evaluated in SPSS Statistics software 24.0 (IBM Corporation, Armonk, NY, USA), and a statistical significance level of 5% was used. Bar graphs represent the means ± standard error of the mean (SE).

## Results

### Effects of LPS and PPi on monocyte adhesion/cell numbers and viability

**Fig. 2:**
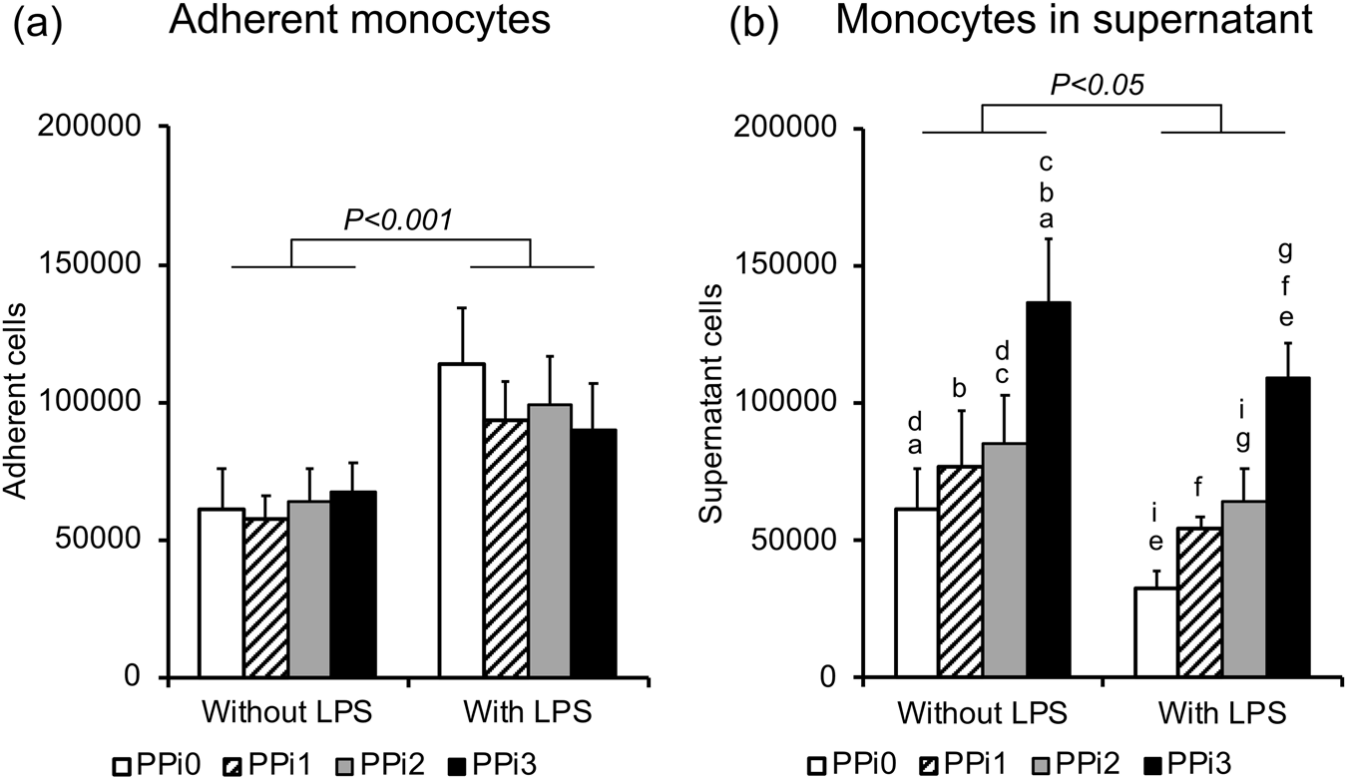
Cell count of monocytes. Cell count of adherent monocytes (**a**) and monocytes in the culture media/supernatant (**b**). Number of monocytes after 48 h of culture in polystyrene plates in the presence or absence of lipopolysaccharide (LPS) and exposed to different concentrations of pyrophosphate (PPi): 0 μM (PPi0), 50 μM (PPi1), 100 μM (PPi2) and 250 μM (PPi3). The bar graphs show the means and standard error of the mean (*n* = 4–5). Connected bars indicate significant differences (*P* < 0.05; *P* < 0.001) between LPS and non-LPS. Small letters indicate significant differences between PPi concentrations (PPi0, PPi1, PPi2, PPi3), where each two similar letters indicate a statistically significant difference (*P* < 0.05) between the two concentrations they are representing

Monocyte adhesion was increased by the presence of LPS (*P* < 0.001) but was not affected by PPi (Fig. 2a; Supplementary Table S1). The number of monocytes in the supernatant was significantly reduced by LPS (20–47% decrease, *P* < 0.05) (Fig. 2b; Online Resource 1 (Supplementary Table S2)) in all PPi groups. For both inflammatory and noninflammatory conditions, the number of supernatant cells increased with increasing PPi concentration, with significant differences for both the PPi2 and PPi3 concentrations (100 and 250 μM) in comparison to cells in the PPi0 group, without the inclusion of PPi (Fig. 2b). Monocyte viability was high (average LDH 0.16 ± 0.02 μKatal/L) and was unaffected by the presence of either PPi or LPS (Online Resource 1 (Supplementary Table S2)).

### Effects of LPS, PPi and condition on MSC adhesion/cell numbers and viability

**Fig. 3:**
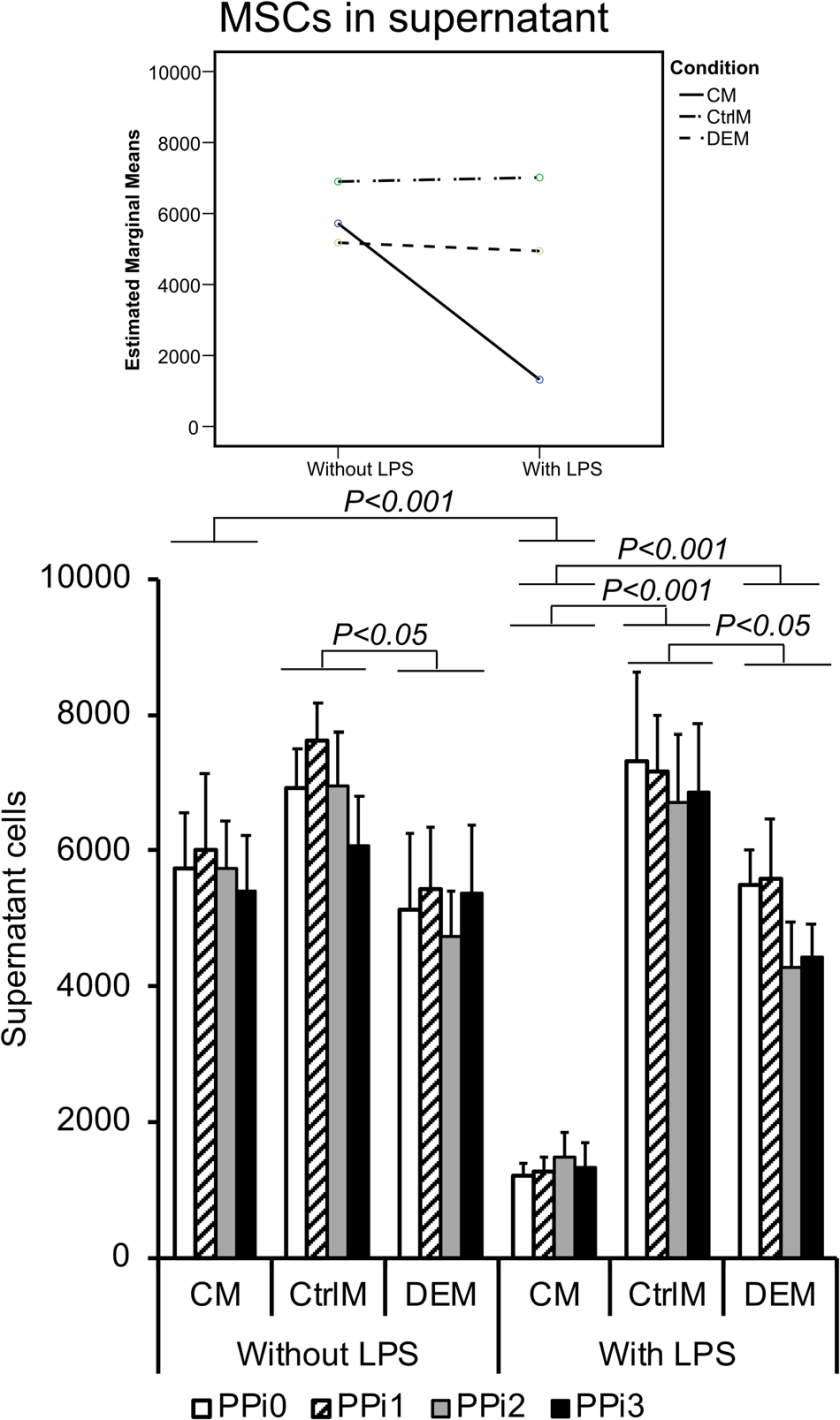
Cell count of mesenchymal stem cells (MSCs). Number of MSCs in the supernatant after 72 h of culture in different culture media: CM = conditioned media from monocytes incubated for 48 h in polystyrene well plates in the presence or absence of lipopolysaccharide (LPS) and exposed to different pyrophosphate (PPi) concentrations; CtrlM = control media incubated for 48 h in polystyrene well plates in the presence or absence of LPS and exposed to different PPi concentrations; DEM = direct effect media incubated for 48 h in 50 mL tubes and used for direct exposure of MSCs to LPS and different PPi concentrations. The different PPi concentrations were as follows: 0 μM (PPi0), 50 μM (PPi1), 100 μM (PPi2) and 250 μM (PPi3). The line graph (top) shows the interaction effect of culture condition and exposure to LPS, whereas the bar graph (bottom) shows the mean and standard error of the mean (*n* = 4–5). Connected bars indicate significant differences (*P* < 0.05; *P* < 0.001) between LPS and non-LPS as well as between the different conditions (CM, CtrlM, DEM)

The amount of adherent MSCs in the wells after 72 h of incubation in the assigned media was similar between all conditions and inflammatory status and was not affected by PPi (Online Resource 1 (Supplementary Table S2)). However, the number of nonadherent MSCs (making up approximately 10% of the total number of cells) was significantly reduced in the CM from LPS-stimulated monocytes versus CM from nonstimulated monocytes (74–79% decrease, *p* < 0.001) (Fig. 3; Online Resource 1 (Supplementary Table S2)).

**Fig. 4:**
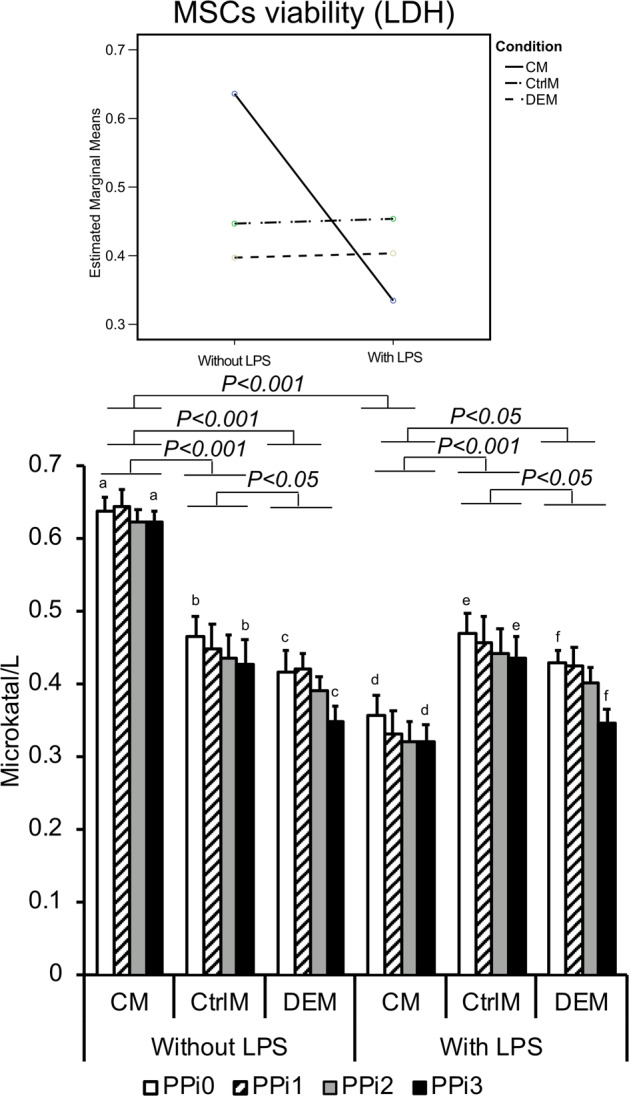
Viability of mesenchymal stem cells (MSCs). The viability of MSCs was measured by lactate dehydrogenase (LDH) analysis. The LDH enzyme activity in the media of MSCs after 72 h of culture in different culture media: CM = conditioned media from monocytes incubated for 48 h in polystyrene well-plates in the presence or absence of lipopolysaccharide (LPS) and exposed to different pyrophosphate (PPi) concentrations; CtrlM = control media incubated for 48 h in polystyrene well-plates in the presence or absence of LPS and exposed to different PPi concentrations; DEM = direct effect media incubated for 48 h in 50 mL tubes and used for direct exposure of MSCs to LPS and different PPi concentrations. The different PPi concentrations were as follows: 0 μM (PPi0), 50 μM (PPi1), 100 μM (PPi2) and 250 μM (PPi3). The line graph (top) shows the interaction effect of culture condition and exposure to LPS, whereas the bar graph (bottom) shows the mean and standard error of the mean (*n* = 4–5). Connected bars indicate significant differences (*P* < 0.05; *P* < 0.001) between LPS and non-LPS as well as between the different conditions (CM, CtrlM, DEM). Small letters indicate significant differences between PPi concentrations (PPi0, PPi1, PPi2, PPi3), where each two similar letters indicate a statistically significant difference (*P* < 0.05) between the two concentrations they are representing

The average MSC viability ranged from 0.33 to 0.64 μKatal/L for the different conditions with or without LPS, with the highest viability among cells in the monocyte-conditioned media with LPS and the lowest viability for MSCs in monocyte-conditioned media without LPS. The highest concentration of PPi (250 μM) resulted in significantly higher MSC viability compared with 0 μM PPi in all culture conditions (2–16% without LPS and 10–9% with LPS; *P* < 0.05 across the groups), although this difference was more pronounced for cells in direct contact with PPi, i.e., DEM (Fig. 4).

### Effects of LPS, PPi and condition on MSC gene expression

#### Proliferation- and death-related genes

MSC expression of the proliferation-related genes *PCNA* and *KI67* and the cell death-related genes *CASP3* and *P53* did not show any dependence upon PPi concentration, inflammatory environment, or condition in the model used (Online Resource 1 (Supplementary Table S2)).

#### Bone-related genes

**Fig. 5:**
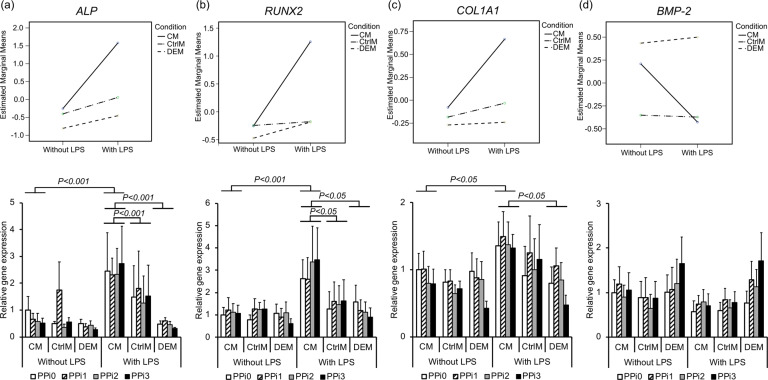
Gene expression of bone-related factors in mesenchymal stem cells (MSCs). Gene expression analysis of (**a**) alkaline phosphatase (*ALP*), (**b**) runt-related transcription factor 2 (*RUNX2*), (**c**) collagen 1 alpha 1 (*COL1A1*) and (**d**) bone morphogenetic protein 2 (*BMP-2*) in MSCs after 72 h of culture in different culture media: CM = conditioned media from monocytes incubated for 48 h in polystyrene well plates in the presence or absence of lipopolysaccharide (LPS) and exposed to different pyrophosphate (PPi) concentrations; CtrlM = control media incubated for 48 h in polystyrene well plates in the presence or absence of LPS and exposed to different PPi concentrations; DEM = direct effect media incubated for 48 h in 50 mL tubes and used for direct exposure of MSCs to LPS and different PPi concentrations. The different PPi concentrations were as follows: 0 μM (PPi0), 50 μM (PPi1), 100 μM (PPi2) and 250 μM (PPi3). The line graph (top) shows the interaction effect of culture condition and exposure to LPS, whereas the bar graph (bottom) shows the mean and standard error of the mean (*n* = 4–5). Connected bars indicate significant differences (*P* < 0.05; *P* < 0.001) between LPS and non-LPS as well as between the different conditions (CM, CtrlM, DEM)

Following exposure to 24 different test conditions, distinct gene expression patterns were observed in MSCs. For bone-related genes, LPS exposure and condition, as well as the interaction effect between the two, were of importance, whereas the PPi concentration did not significantly affect gene expression (Online Resource 1 (Supplementary Table S2)). The key osteogenic commitment transcription factor *RUNX2* revealed 2.6- (PPi0), 2- (PPi1), 3- (PPi2) and 3.5-fold (PPi3) significantly higher expression in MSCs cultured in CM from LPS-stimulated monocytes than in MSCs cultured in nonstimulated monocyte CM (*P* < 0.05 across the material groups) (Fig. 5b). Similarly, the early osteogenic differentiation marker ALP showed 2.5- (PPi0), 3.5- (PPi1), 4- (PPi2) and 5-fold (PPi3) significant upregulation in MSCs cultured in CM from LPS-stimulated monocytes compared to nonstimulated monocytes (*P* < 0.001 across the material groups) (Fig. 5a). A similar pattern was seen for *COL1A1* (Fig. 5c), although the effect of LPS-stimulated monocyte CM did not reach statistical significance in the MANOVA test. In contrast, no major effect was observed for LPS-stimulated monocyte CM on the MSC expression of BMP-2 (Fig. 5d). For BMP2, a 2.5-fold higher trend of expression was observed in MSCs directly exposed to the highest PPi concentration (PPi3) in the DEM (with and without LPS) compared to PPi3 in control media (CtrM) or to no-PPi (PPi0) in direct effect media (DEM) (Fig. 5d). Other bone-related genes, such as *OPN* and *TGF-β*, were not affected by any of the investigated parameters (*P* > 0.05, Online Resource 1 (Supplementary Table S2)).

#### Cartilage- and adipose-related genes

**Fig. 6:**
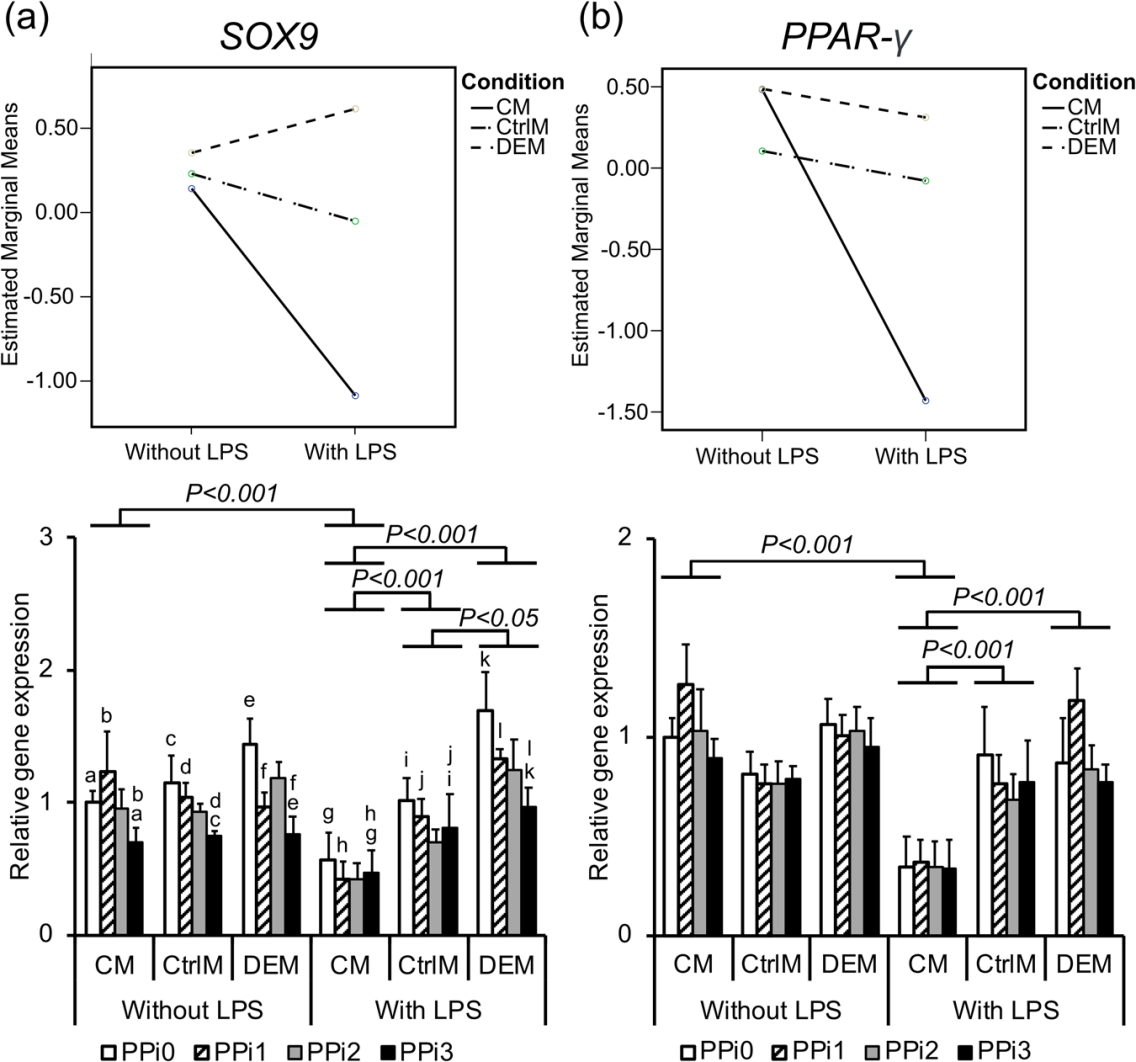
Gene expression of chondrogenic and adipogenic factors in mesenchymal stem cells (MSCs). Gene expression analysis of (**a**) chondrogenic transcription factor SRY-box 9 (*SOX9*) and (**b**) adipogenic transcription factor peroxisome proliferator-activated receptor gamma (*PPAR-γ*) in MSCs after 72 h of culture in different culture media: CM = conditioned media from monocytes incubated for 48 h in polystyrene well-plates in the presence or absence of lipopolysaccharide (LPS) and exposed to different pyrophosphate (PPi) concentrations; CtrlM = control media incubated for 48 h in polystyrene well-plates in the presence or absence of LPS and exposed to different PPi concentrations; DEM = direct effect media incubated for 48 h in 50 mL tubes and used for direct exposure of MSCs to LPS and different PPi concentrations. The different PPi concentrations were as follows: 0 μM (PPi0), 50 μM (PPi1), 100 μM (PPi2) and 250 μM (PPi3). The line graph (top) shows the interaction effect of culture condition and exposure to LPS, whereas the bar graph (bottom) shows the mean and standard error of the mean (*n* = 4–5). Connected bars indicate significant differences (*P* < 0.05; *P* < 0.001) between LPS and non-LPS as well as between the different conditions (CM, CtrlM, DEM). Small letters indicate significant differences between PPi concentrations (PPi0, PPi1, PPi2, PPi3), where every two similar letters indicate a statistically significant difference (*P* < 0.05) between the two concentrations they are representing

A significant downregulation in cartilage-related gene expression, *SOX9*, was revealed when MSCs were exposed to inflammatory monocyte-conditioned media (Fig. 6a). Moreover, the presence of PPi resulted in downregulation of *SOX9* gene expression in a concentration-dependent manner, with a significant difference between PPi3 and PPi0 (1.2- to 2-fold downregulation, *P* < 0.05 across groups) and between PPi3 and PPi1 (1.2- to 2-fold downregulation, *P* < 0.05 across groups) (Fig. 6a). Additionally, the gene expression of *PPAR-γ*, which regulates the differentiation of MSCs into adipocytes, was significantly downregulated 3- to 3.5-fold when MSCs were exposed to inflammatory monocyte-conditioned media compared to nonstimulated monocyte CM (*P* < 0.001 across the material groups) (Fig. 6b).

## Discussion

The present study investigated the role of monocyte-to-MSC communication via soluble factors on bone differentiation in inflammatory and noninflammatory environments in vitro. Furthermore, the effects of pyrophosphate on MSC differentiation, as well as monocyte and MSC survival, were addressed.

### Effects of LPS and PPi on monocyte adhesion/cell numbers and viability

Bacterial stimuli (LPS) had a profound effect on the distribution of monocytes, resulting in higher cell adhesion and a lower number of monocytes in suspension. A similar effect has been observed elsewhere and attributed to reduced caspase activity and increased Akt phosphorylation [35]. In contrast to monocytes, the amount and distribution of MSCs were not affected by direct exposure to LPS. However, the amount of MSCs in the supernatant of conditioned medium obtained from LPS-primed inflammatory monocytes decreased considerably. It is likely that the conditioned media contained inflammatory factors secreted from monocytes exposed to LPS, which promoted MSC viability and adhesion. Interestingly, MSC viability increased significantly in LPS-monocyte-conditioned media. Thus, LPS-stimulated monocytes appear to produce survival factor(s) that improve MSC viability. In contrast, MSCs incubated in noninflammatory monocyte-conditioned media (-LPS) exhibited significantly lower viability. It is possible that the lower MSC viability in monocyte-conditioned medium (CM) compared to CtrM and DEM is due to the low starting media serum levels (1%), which were further reduced during the 48 h incubation with monocytes. Taking into account the serum depletion occurring in all CM groups, the observed increase in MSC viability in LPS-primed monocyte CM appears even more potent.

### PPi increases the survival of primary human monocytes and MSCs

In the present study, cells were exposed to pyrophosphate, both as ions in solution (sodium pyrophosphate) in all three conditions and as precipitants (in the form of amorphous calcium pyrophosphate precipitant) [15] in monocyte cultures and under DEM conditions for MSCs. In monocyte cultures, the presence of high PPi concentrations (250 μM) significantly increased the number of monocytes in suspension without affecting the number of adherent monocytes. Thus, monocytes exhibited a survival effect from PPi at low-serum levels (1%), similar to previous reports in fibroblasts [36]. MSC viability (LDH) was higher following exposure to 250 μM PPi than following exposure to 0 PPi, further supporting a survival factor-like effect of PPi in multiple cell types. The increase in cell viability was particularly apparent under DEM conditions, implicating the PPi-precipitates – present in both monocytic- and DEM-cultures – as responsible for this effect. PPi precipitants require direct contact with the cell membrane [37], as the survival effects of pyrophosphate are abolished if cell cultures are inverted (grown on the ceiling of the culture well), thus indicating that it is the interaction between the PPi crystals and the cell surface that is of importance for increased cell survival. Interestingly, other calcium-containing, basic calcium phosphate (BCP) particles produce survival effects in macrophages [38]. Furthermore, calcium pyrophosphate, known to have low solubility and dissolution properties, produces similar responses in inflammatory cells as BCPs [15].

This is the first study to report that pyrophosphate can act as a survival factor for human MSCs and primary monocytes. Prior studies have investigated the effect of pyrophosphate on the survival of connective tissue cells (fibroblasts) [36] and osteoblasts [15]. The physiological impact of this finding is that PPi may prolong the survival of recruited monocytes, thereby altering both the duration and magnitude of inflammatory signaling. These observations indicate that the role of monocytes and macrophages during bone regeneration induced by pyrophosphate-containing bone ceramics [16, 17, 28] should be explored.

### LPS and monocyte-conditioned media promote osteogenic differentiation in MSCs

A key observation was that monocytes play a role in the differentiation of MSCs, preferentially in inflammatory conditions. The gene expression of *RUNX2*, *ALP* and *COL1A1* was significantly upregulated in MSCs that were exposed to monocyte-conditioned media (+LPS). The expression of these three genes is necessary for bone cell differentiation and bone tissue formation. In addition, both the cartilage- and adipose-cell differentiation pathways (*SOX9* and *PPAR-γ*) were downregulated in response to monocyte-conditioned media (+LPS).

Monocytes, which differentiate into macrophages, are known to produce multiple cytokines, chemokines, growth factors, and other mediators of inflammation. Macrophages are key players in orchestrating the cascade of events that ultimately resolve inflammation and promote tissue healing. In response to infection (or inflammation), primed monocytes produce factors that direct MSCs towards the bone lineage. In agreement with our findings in vitro, infection and inflammation can potentiate bone formation in vivo [10, 39, 40] Mineralized tissue formation, specifically at the material/tissue interface in vivo, is significantly increased when the implants are preincubated in LPS or when monocytes are selectively polarized (M1, M2) compared to controls [10, 39–41].

The exposure timing, when monocytes sense and react to the environment, is also of importance. Unactivated and M1 macrophages stimulate osteogenic differentiation of MSCs only during the early stages (1–7 days), whereas M2 polarized macrophages stimulate proliferation and differentiation, including matrix proteins such as osteocalcin and bone sialoprotein, more strongly at late (7–14 days) but not early time points (1–7 days) [39]. Monocytes incubated with LPS, or polarized directly to M1, also secrete molecules that promote increased BMP-2 gene expression in human MSCs [10, 42]. In the present study, a trend for upregulation of BMP-2 gene expression in MSCs was observed only in response to PPi exposure and not in response to monocyte-conditioned media, regardless of the presence of LPS.

### PPi downregulate SOX9 expression in MSCs

Interestingly, in MSCs, the expression of only one of the genes analyzed was dependent upon the PPi concentration: *SOX9*. The expression of *SOX9* decreased in response to PPi in a concentration-dependent manner. The *SOX9* gene is involved in the chondrogenic differentiation of MSCs [43]. Reduced *SOX9* expression is implicated in excessive/ectopic mineralization, whereas overexpression of *SOX9* is associated with increased chondrogenesis and reduced osteogenesis [43–45]. The concomitant reduction in *SOX9* and *PPAR-γ* gene expression strongly favors the differentiation of MSCs towards osteogenic lineages rather than adipogenic or chondrogenic lineages, suggesting that the osteogenic effects of PPi may be due, in part, to a decrease in adipogenic and chondrogenic differentiation signaling. Interestingly, *PPAR-γ* expression and phosphorylation are related to MSC serum levels [46]. Since pyrophosphate can act as a survival factor in low-serum conditions [36], it is possible that pyrophosphate affects gene expression via serum sensing/signaling [15].

The expression of several genes was affected by PPi concentration (although not significantly), particularly following direct exposure to pyrophosphate solution/precipitant (DEM). *RUNX2* gene expression was negatively affected by exposure to pyrophosphate, whereas expression of the *COL1A1*, *ALP* and *TGF-β* genes was elevated at lower concentrations of PPi but then reduced in a concentration-dependent manner when the PPi concentration was increased. In contrast, *BMP-2* expression was increased in a concentration-dependent manner when exposed to PPi but only in the DEM group. BMP-2 is a strong inducer of osteogenic differentiation and bone tissue formation, and the apparent concentration-dependent increase in expression in response to pyrophosphate under direct exposure conditions (DEM) further supports the notion that pyrophosphate can elicit osteogenic expression in MSCs.

In prior studies, a biphasic effect on ALP activity and gene expression was observed in preosteoblasts, whereby 100 nM sodium pyrophosphate stimulated greater osteogenic gene expression than 100 μM [15]. In the present study, lower concentrations of pyrophosphate also stimulated higher osteogenic gene expression in MSCs (DEM). The optimal concentration in the present study (50 μM) was just below the concentration at which sodium pyrophosphate precipitates as calcium pyrophosphate, whereas in osteoblasts, the optimal concentration was much lower (100 nM) [15].

To the authors’ knowledge, this is the first study to examine the effect of pyrophosphate on cell-cell communication. Taken together, the results of the present work suggest that pyrophosphate directly stimulates MSCs towards an osteogenic, bone-forming lineage while also discouraging differentiation into fat- and cartilage lineages, but only when in direct contact with crystals/precipitants. These findings are particularly significant because prior studies have focused solely on single cell types or single pathophysiologies (i.e., osteoblast-mediated mineralization or pseudogout), rather than on how pyrophosphate stimulates intercellular communication, leading to inflammatory cell polarization [13], cellular signaling between different cell types [47], and osteogenic differentiation [15].

The findings of the present study refute the underlying null-hypothesis, and show that that PPi conveys direct effects on monocytes and MSCs, increasing their survival. Moreover, the data revealed significant effect of PPi on regulating the early gene expression of MSCs, favoring the downregulation of *SOX9* gene that drives the MSCs toward the chondrogenic lineage. Moreover, the present study extends and corroborates previous observations [10] that factors secreted from LPS-stimulated pro-inflammatory macrophages act as potent stimuli for early osteogenic gene expression in the recipient MSCs. The latter finding is also of clinical interest, emphasizing the important role of the initial inflammatory response after surgery and material implantation for triggering the subsequent regenerative processes. In addition, there is mounting evidence that calcium pyrophosphate can enhance bone formation in vivo when mixed with calcium phosphate cement [16, 28]. We have demonstrated two mechanisms that may contribute to this phenomenon. Pyrophosphate, as an additive or coating, may tentatively improve the biological response to other bioceramic implants. If proven, this would be important for clinical translation, enabling the production and control of the properties of inorganic additives.

It should be noted that a) the media for direct and indirect exposure to pyrophosphate lacked osteogenic factors, such as dexamethasone, ascorbic acid, or beta-glycerol phosphate; and b) the selected time points in the present study were relatively early, excluding observations at later but still relevant time stages for osteogenic differentiation. Without osteogenic media, multipotent cells typically exhibit much higher stochastic “noise,” and the absolute magnitude of the cellular response (i.e., changes in gene expression) to external stimulants (i.e., different surface chemistry) is often quite weak compared to cultures that include osteogenic media. Subsequent studies should investigate how PPi affects MSC differentiation under osteogenic conditions, where effects are likely to occur at much greater magnitudes.

## Conclusions

In conclusion, the results of the present in vitro study showed that high concentrations of pyrophosphate improved monocyte and MSC survival. Furthermore, the inflammatory environment, including monocyte-MSC paracrine communication, affected the early differentiation of MSCs towards osteogenesis. Calcium pyrophosphate precipitants stimulated pro-osteogenic gene expression. Inflammatory monocytes appear to skew MSCs towards the osteogenic lineage by (1) potentiating MSCs to early differentiation into bone cells via increased expression of *RUNX2, ALP* and *COL1A1* and (2) hindering MSC differentiation towards the cartilage and adipocyte lineages, as revealed by suppressed *SOX9* and *PPAR-γ* expression.

## Supplementary Information

Electronic Supplementary Material ESM_1
