# Supplementary material for: Monocytes and pyrophosphate promote mesenchymal stem cell viability and early osteogenic differentiation
Source: J Mater Sci Mater Med. 2022 Jan 15;33(1):11. doi: 10.1007/s10856-021-06639-y (PMC8761140; doi:10.1007/s10856-021-06639-y)
Supplement: Supplementary file 1 — Electronic Supplementary Material ESM_1 [file 10856_2021_6639_MOESM1_ESM.pdf]

Monocytes and pyrophosphate promote mesenchymal stem cell viability and early osteogenic differentiation

Journal of Materials Science: Materials in Medicine

Sara Svensson[1], Michael Palmer[2], Johan Svensson[3], Anna Johansson[1], Håkan Engqvist[2], Omar Omar[4] and Peter Thomsen[1,*]
[1]Department of Biomaterials, Institute of Clinical Sciences, Sahlgrenska Academy, University of Gothenburg, Gothenburg, Sweden
[2]Department of Engineering Sciences, Applied Materials Science Section, Uppsala University, Uppsala, Sweden
[3]Department of Statistics, Umeå School of Business, Economics and Statistics, Umeå University, Umeå, Sweden
[4]Department of Biomedical Dental Sciences, College of Dentistry, Imam Abdulrahman bin Faisal University, Dammam, Saudi Arabia

***Correspondence**
Peter Thomsen, MD, PhD, Professor
Department of Biomaterials, Institute of Clinical Sciences, Sahlgrenska Academy at the University of Gothenburg, Box 412, SE-405 30 Gothenburg, Sweden
Telephone: + 46 (0) 705255554
Email: peter.thomsen@biomaterials.gu.se

Reference gene screening

Screening for best stable reference gene(s) was conducted on a panel of selected 8 human reference genes (TATAA Biocenter AB, Gothenburg, Sweden) (Table S1), in accordance with the MIQE guidelines for the performance and reporting of the gene expression analysis [1]. The Cq values from the qPCR screening assays were subsequently analyzed in GenEx software version 6 (MultiD Analyses AB, Gothenburg, Sweden), using both the geNorm and NormFinder algorithms. Based on this analysis, a single reference gene was recommended for normalization, and the tyrosine 3-monooxygenase/tryptophan 5-monooxygenase activation protein zeta (YWHAZ) was identified as the best stable reference gene, and, therefore selected for normalization.

1. Bustin, S. A., Benes, V., Garson, J. A., Hellemans, J., Huggett, J., Kubista, M., Mueller, R., Nolan, T., Pfaffl, M. W., Shipley, G. L., Vandesompele, J. & Wittwer, C. T. The miqe guidelines: Minimum information for publication of quantitative re-al-time pcr experiments. Clin Chem 2009;55:611-622.

Table S1: List of reference genes used for the screening and selection of best stable reference gene(s) for normalization.

**Abbreviation**	**Full name (according to NCBI database)**
YWHAZ	Tyrosine 3-monooxygenase/tryptophan 5-monooxygenase activation protein zeta
PPIA	Peptidylprolyl isomerase A
B2M	Beta-2-microglobulin
HPRT1	Hypoxanthine phosphoribosyltransferase 1
TBP	TATA-box binding protein
RPLP	50S ribosomal subunit protein L16
UBC	Ubiquitin C
18S	Ribosomal protein S18

Statistical analysis

Due to the large number of related response variables in the experiment, the statistical analyses were carried out using multivariate analysis of variance (MANOVA). Here, the responses were divided into six different groups: 1) monocyte cell numbers and cell viability (three responses), 2) MSC cell numbers and cell viability (three responses), 3) MSC bone-related gene expression (six responses), 4) MSC proliferation-related gene expression (two responses), 5) MSC death-related gene expression (two responses), and 6) MSC fat- and cartilage-related genes (two responses). For the group of monocyte response variables, a MANOVA with the factors LPS (two levels; with and without LPS) and Materials (four levels; PPi0, PPi1, PPi2 and PPi3) was performed. For the five groups of MSC response variables, additional two factors were included in the MANOVA: Condition (three levels; CM, CtrlM and DEM) and interaction between LPS and Condition.

The Wilks' λ test statistic assesses if the factors relate to any of the responses. If so, the relationships between individual factors and responses were further investigated by ANOVAs (Table S2). This approach protects against false-positive results related to mass significance. For evaluation of the difference in response between factor levels, the estimated marginal means with Bonferroni correction was used (presented in the figures).

Table S2: Multivariate analyses of variances (MANOVAs) for monocyte and mesenchymal stem cell (MSC) cell number and viability as well as the gene expression in MSCs (Tests of Between-Subjects Effects).

MANOVAS			Factors (*P-value*)				$R^{2\#}$
			**LPS**	**Condition**	**Material**	**LPS×Condition**	
Monocytes number and viability	Wilks' λ*		<0.001	N.A.	0.003	N.A.	
	Response	Adherent cells	0.001	N.A.	0.858	N.A.	28%
		Supernatant cells$^{\Delta}$	0.022	N.A.	<0.001	N.A.	50%
		Viability	0.078	N.A.	0.951	N.A.	9%
MSC number and viability	Wilks' λ		<0.001	<0.001	0.284	<0.001	
	Response	Adherent cells$^{\Delta}$	0.889	0.074	0.728	0.58	7%
		Supernatant cells	<0.001	<0.001	0.48	<0.001	62%
		Viability	<0.001	<0.001	0.021	<0.001	77%
Bone-related gene expression	Wilks' λ		<0.001	<0.001	0.15	0.003	
	Response	*ALP*	<0.001	<0.001	0.463	0.016	32%
		BMP-2	0.443	0.035	0.554	0.468	10%
		COL1A1	0.079	0.031	0.281	0.196	15%
		RUNX2	0.013	0.013	0.967	0.034	18%
		OPN	0.127	0.757	0.372	0.162	8%
		TGF-β	0.251	0.748	0.349	0.587	6%
Proliferation-related genes	Wilks' λ		0.481	0.933	0.516	0.309	
	Response	*KI67*$^{\Delta}$	0.228	0.844	0.546	0.369	6%
		PCNA	0.841	0.809	0.395	0.225	6%
Death-related genes	Wilks' λ		0.075	0.025	0.614	0.006	
	Response	*CASP3*	0.433	0.386	0.347	0.005	14%
		P53	0.31	0.348	0.364	0.685	7%
Fat & cartilage-related genes	Wilks' λ		<0.001	<0.001	0.008	<0.001	
	Response	*PPAR-γ*$^{\Delta}$	<0.001	<0.001	0.637	<0.001	49%
		SOX9$^{\Delta}$	<0.001	<0.001	0.007	<0.001	50%

* = Multivariate test statistic related to MANOVA.

# = Coefficient of determination.

Δ = Sensitivity analysis using bootstrap is presented since the homoelasticity assumption was doubtful.
